# Effects of High Versus Low Flux Membranes on O2 Saturation in Hemodialysis Patients

**DOI:** 10.5812/numonthly.5055

**Published:** 2012-12-15

**Authors:** Ali Momeni, Hamid Rouhi, Masoud Amiri

**Affiliations:** 1 Department of Internal Medicine, Shahrekord University of Medical Sciences, Shahrekord, IR Iran; 2 Social Health Determinants Research Center and Department of Epidemiology and Biostatistics, School of Health, Shahrekord University of Medical Sciences, Shahrekord, IR Iran

**Keywords:** Membranes, Oxymetry, Renal Dialysis

## Abstract

**Background:**

Several studies have been carried out to evaluate the effects of dialysis on O2 saturation. While the dialysis procedure may lead to hypoxia under different circumstances, there are few studies available on the effects of membrane type on O2 saturation in these patients.

**objectives:**

This study was to appraise the effects of high and low flux membrane on pulse oxymetery in dialysis patients.

**Patients and Methods:**

In a cross-sectional evaluation, 43 hemodialysis patients without pulmonary disease were enrolled. Of this group, dialysis was performed by low and high flux membranes, and pulse oxymetery was applied before and after the procedures.

**Results:**

Mean age of the patients was 56.34 years. Of these patients, 23 (53.5%) and 20 (46.5%) were women and men, respectively. Type of membrane (high flux vs. low flux) did not show any significant effect on pulse oxymetery results (*P* > 0.05).

**Conclusions:**

Due to the lack of a significant difference in pulse oxymetery and creation of hypoxia between two types of membranes in hemodialysis patients, as well as the high cost of high flux membrane as compared to the low flux membrane, we do not suggest the use of high flux membrane in dialysis.

## 1. Background

Low flux or high flux membranes can be applied for hemodialysis. High-flux dialysis is defined as a β2-microglobulin clearance of over 20 mL/min (1, 2). High flux membranes compared to low flux have larger pores and allow the diffusing of a greater amount of uremic toxins and middle molecules such as β2-microglobuline and may, therefore, decrease the risk of dialysis-related amyloidosis (3, 4). In addition, these membranes have other advantages such as increasing patients’ survival (5, 6), reduced admission and morbidity (7, 8), fewer activations of the coagulation pathway and complement system, lower leukocytosis, fewer activations of inflammatory system and less cytokines secretion, removal of more endotoxines, better lipid profile (9, 10), reduced infection risk, aluminum toxicity and better preserved renal function (11, 12).

Furthermore, intradialysis hypoxia was reported in former studies (13-15). Although the cause of this hypoxia is not completely clear, sequestration of leukocytes in pulmonary capillary may play an important role (16). The type of buffer used in dialysis also may be important in hypoxia (17, 18).

## 2. Objectives

As far as our knowledge, there is no study about the relationship between the type of hemodialysis membrane (high flux versus low flux) and hypoxia. Since high flux membranes are more permeable than low flux membrane, they might be responsible for the changes in O2 pressure and saturation in a dialysis session. The objective of our study was the evaluation of this relationship.

## 3. Patients and Methods

In a cross-sectional study, 43 volunteer hemodialysis patients were enrolled from June to December 2009 at Hajar hospital in Shahrekord, Iran. Inclusion criteria were age more than 18 years and duration of dialysis more than 6 months. Exclusion criteria were individuals who were non-cooperation during study, history of pulmonary disease and need for oxygen during dialysis.

The dialysis was performed using two types of membranes. While pulse oxymetery performed before and after dialysis with a low flux membrane, in the next dialysis session, pulse oxymetery was repeated in the same patients with a high flux membrane. Pulse oxymetery was done by Mir instrument (Italy). Dialysis by Ferezinius and Gambro digital machines (USA), 2 to 3 times per week, as a regular method (4 hours with blood flow (QB) of 250 to 350 mL/min, dialysate flow (QD) of 500 ml/min and ultra filtration based on the patients' condition) had been performed. Chi-square and t-student tests according to quantitative of qualitative nature of variables, and also Pearson correlation to find our potential correlations, by using SPSS (version 19) had been used.

## 4. Results

Table 1 summarizes the results of our study. Out of 43 dialysis patients, twenty three were women (53.5%). The causes of renal failure were diabetes mellitus, hypertension, hereditary kidney disease and proteinuria in 23, 14, 3, and 3 patients, respectively. Ischemic heart disease was observed in 23 patients and three cases had congestive heart failure. Mean age of the patients was 56.34 years (ranged 23 to 84). While the weight of the patients before and after dialysis with a low flux membrane were 62.5 and 59.8 kilograms respectively (*P* < 0.001), the weight of patients before and after dialysis with a high flux membrane was 61.9 and 59.5 kilograms respectively (*P* < 0.001).

**Table 1:** Comparison of Variables of Patients With High and Low Flux Membrane After Dialysis

	Membrane Type	Mean	Standard Deviation	*P*
**Body weight before dialysis, kg**				0.8
	Low flux	62.5	12.3	
	High flux	61.9	12.5	
**Body weight after dialysis, kg**				0.9
	Low flux	59.8	12.1	
	High flux	59.5	12.1	
**Diastolic blood pressure before dialysis, mmHg**				0.15
	Low flux	73.1	9.1	
	High flux	75.9	8	
**Diastolic blood pressure after dialysis, mmHg**				0.7
	Low flux	73.9	9	
	High flux	73.2	9.1	
**Systolic blood pressure before dialysis, mmHg**				0.3
	Low flux	125.5	11.5	
	High flux	129.5	12.5	
**Systolic blood pressure after dialysis, mmHg**				0.7
	Low flux	121.3	15.8	
	High flux	119	9.8	
**O2 saturation before dialysis**				0.3
	Low flux	93.9	2.6	
	High flux	95	3.8	
**O2 saturation after dialysis**				0.1
	Low flux	93.2	3.7	
	High flux	93.4	2.9	

Blood pressure before and after dialysis with a low flux or a high flux membrane did not show statistically significant differences (*P* > 0.05). O2 saturation before dialysis with a low flux membrane was 92.3% and after dialysis was 93.2% (*P* = 0.2). Similarly, O2 saturation before and after dialysis with a high flux membrane was 95% and 94.4% respectively (*P* = 0.5). There was also no difference between O2 saturation with high flux and low flux membranes, before or after dialysis (*P* > 0.05).

## 5. Discussion

To our knowledge, this study is the first investigation on the relationship between types of hemodialysis membrane (high flux versus low flux) and hypoxia. There was no difference between oxygen saturation in hemodialysis’ patients with high flux and low flux membranes. Based on our results, there was not any advantage to the high versus the low flux membrane.

Several studies were conducted on hypoxia due to hemodialysis and its physiopathology (15-17), such as Habte (15) who reported hypoxia of the patients during dialysis, Kao (19) with reporting of decreasing permeability of respiratory membrane to oxygen but disappearing after dialysis. However, Dhakal suggested that intradialysis hypoxia could be continued even after dialysis (20). Yap also concluded that intra dialytic oxygen didn’t cause improvement of ventilation profile (21). Furthermore, Hunt reported that intradialytic hypoxia depended on the type of buffer in the dialysis procedure so if acetate was used as a buffer, the oxygen removed from dialysate and hypocapnia caused depression of the respiratory center and mild hypoxia, however, the increasing serum bicarbonate and central depression occurred in the bicarbonate buffer (15, 19). Pitcher also reported equivalent results and mild hypoxia especially in patients with respiratory disease (14).

The other factor that may cause hypoxia in dialysis is sequestration of leukocytes in pulmonary capillaries during the early part of the dialysis procedure. This effect was reported by Craddock and Fountain (17, 22). There are different findings about the relationship of leucopenia and hypoxia during dialysis. For example, Ralph showed leucopenia without hypoxia at beginning of a dialysis session. This leukopenia disappeared after 2 hours and may have been accompanied with reactive leukocytosis (23). Fawcett and Vaziri in their studies reported less hypoxia with biocompatible membranes versus old membranes (24, 25). Unlike the above results, Hakim showed that hypoxia during hemodialysis was not significant and that there was no difference between the use of cynthetic or cellulose membrane in this respect (26).

In conclusion, our results indicated that membrane type (high vs. low flux) has no effect on the development or severity of intradialysis hypoxia. Routinely, dialysis procedure in Hajar hospital is performed with low flux membrane. If a high flux membrane had been used for a longer period, pulse oxymetery results may have been different from patients with a low flux membrane therefore we do recommend larger and longer follow up studies with high flux membrane in hemodialysis patients.
